# *Toxoplasma gondii* dense granule protein GRA24 drives MyD88-independent p38 MAPK activation, IL-12 production and induction of protective immunity

**DOI:** 10.1371/journal.ppat.1008572

**Published:** 2020-05-15

**Authors:** Heather L. Mercer, Lindsay M. Snyder, Claire M. Doherty, Barbara A. Fox, David J. Bzik, Eric Y. Denkers

**Affiliations:** 1 Center for Evolutionary and Theoretical Immunology and Department of Biology, University of New Mexico, Albuquerque, New Mexico, United States of America; 2 Department of Microbiology and Immunology, Geisel School of Medicine at Dartmouth, Lebanon, New Hampshire, United States of America; University of Massachusetts Medical School, UNITED STATES

## Abstract

The apicomplexan *Toxoplasma gondii* induces strong protective immunity dependent upon recognition by Toll-like receptors (TLR)11 and 12 operating in conjunction with MyD88 in the murine host. However, TLR11 and 12 proteins are not present in humans, inspiring us to investigate MyD88-independent pathways of resistance. Using bicistronic IL-12-YFP reporter mice on *MyD88*^*+/+*^ and *MyD88*^*-/-*^ genetic backgrounds, we show that CD11c^+^MHCII^+^F4/80^-^ dendritic cells, F4/80^+^ macrophages, and Ly6G^+^ neutrophils were the dominant cellular sources of IL-12 in both wild type and MyD88 deficient mice after parasite challenge. Parasite dense granule protein GRA24 induces p38 MAPK activation and subsequent IL-12 production in host macrophages. We show that *Toxoplasma* triggers an early and late p38 MAPK phosphorylation response in *MyD88*^*+/+*^ and *MyD88*^*-/-*^ bone marrow-derived macrophages. Using the uracil auxotrophic Type I *T*. *gondii* strain *cps1-1*, we demonstrate that the late response does not require active parasite proliferation, but strictly depends upon GRA24. By i. p. inoculation with *cps1-1 and cps1-1*:*Δgra24*, we identified unique subsets of chemokines and cytokines that were up and downregulated by GRA24. Finally, we demonstrate that *cps1-1* triggers a strong host-protective GRA24-dependent Th1 response in the absence of MyD88. Our data identify GRA24 as a major mediator of p38 MAPK activation, IL-12 induction and protective immunity that operates independently of the TLR/MyD88 cascade.

## Author summary

*Toxoplasma gondii* is a protozoan parasite that infects over 1 billion people worldwide. Infection with the parasite is normally asymptomatic and *Toxoplasma* co-exists with its host in the form of latent cysts in brain and muscle tissue. The balance between immune recognition and immune evasion is likely a key factor in the outcome of this host-parasite interaction. It is therefore important to understand how *Toxoplasma* triggers immunity, and in particular how the protective cytokine IL-12 is induced during infection. While Toll-like receptor (TLR)/MyD88 signaling is important in mouse resistance to *Toxoplasma*, this pathway is likely less important in human infection. Here, we report that the parasite dense granule protein GRA24 triggers p38 MAPK activation and IL-12 production independently of TLR/MyD88 signaling. We identify additional cytokines and chemokines that are regulated by GRA24 during *in vivo* infection. Our data demonstrate that GRA24 initiates a protective MyD88-independent immune response during *in vivo* infection. The GRA24 molecule provides an example of a parasite molecule whose function is induction of a host protective immune response. From the standpoint of *Toxoplasma*, this likely reflects an evolutionary adaptation to ensure host survival and simultaneously enable latency to maximize the chance of transmission.

## Introduction

The intracellular protozoan *Toxoplasma gondii* is a globally distributed parasite that infects humans, companion animals, livestock and wildlife. The parasite is estimated to infect 25–30% of the human population worldwide [1]. The course of infection is characterized by two phases. In the acute phase initiated in the intestine after oral infection, the parasites disseminate widely through tissues as rapidly dividing and highly invasive tachyzoites. This is followed by a chronic, or latent, phase associated with differentiation to slowly dividing bradyzoites that form long-lived cysts in tissues of the skeletal muscle and central nervous system [2]. Infection at this stage is usually asymptomatic. Nevertheless, in immunodeficient populations cysts may reactivate resulting in uncontrolled parasite replication that can rapidly culminate in death [3]. *Toxoplasma* may also cross the placenta during pregnancy causing life-threatening disease both before and after birth [4].

*Toxoplasma* is highly effective at stimulating a protective immune response, an outcome that accounts for the normally asymptomatic nature of infection [5–7]. While clearly aiding the host, the parasite also benefits from this protective response since host survival enables establishment of latent infection. The immune response to *T*. *gondii* revolves around early production of IL-12 by cells such as CD8α dendritic cells (DC) in the spleen as well as CD103^+^CD11b^-^ and CD103^-^CD11b^-^ DC in the intestinal mucosa [8–10]. The central role played by IL-12 in resistance is dramatically highlighted by the extreme susceptibility of IL-12 knockout (KO) mice to *T*. *gondii* infection [11]. Production of IL-12 drives early NK cell production of IFN-γ and generation of IFN-γ-producing Th1 cells. IFN-γ ultimately controls the parasite through its ability to induce anti-*Toxoplasma* effector molecules such as the immunity-related GTPase (IRG) family and guanylate-binding proteins (GBP) that destroy the parasitophorous vacuole harboring intracellular tachyzoites [12–16].

The molecular and cellular basis for recognition and subsequent IL-12 production in response to *T*. *gondii* and other microbial pathogens has been extensively studied in mouse models. Early on, it was understood that the Toll-like receptor (TLR) adaptor molecule MyD88 is important in triggering IL-12 and promoting host resistance to *Toxoplasma* [17,18]. Cell-specific knockout studies revealed CD11c^+^ DC as the major source of MyD88-dependent resistance rather than macrophages or neutrophils that can also contribute to protective IL-12 [19–21]. Amongst TLR, both TLR11 and TLR12 are major receptors involved in recognition and resistance. Together, these receptors are activated by profilin, a parasite protein required for host cell invasion that also serves as a classical pathogen-associated molecular pattern igniting innate immunity [22–27]. Other TLR, for example cell surface TLR2 and 4 and the endosomal nucleic acid receptors TLR7 and 9, have been suggested to play secondary roles in resistance to *Toxoplasma* [24,28,29].

While a great deal is known about initiation of immunity to *T*. *gondii* in mouse models, the major innate immune receptors and signaling molecules involved in human resistance to this intracellular protozoan remains an open question. This is because in humans TLR11 is present only as a pseudogene and the TLR12 gene is entirely absent [30]. Moreover, MyD88-deficient individuals remain resistant to all but a select few pyogenic bacterial infections [31,32]. Thus, while TLR/MyD88 signaling plays a major role in rodent resistance to *Toxoplasma* and other infections, this signaling axis appears less crucial for defense in humans.

Previously, we reported the presence of MyD88-independent pathways of immunity during *Toxoplasma* infection [33]. While MyD88 KO mice ultimately do not survive infection, the days immediately preceding death are characterized by emergence of IFN-γ-producing Th1 effectors. More strikingly, vaccination of *MyD88*^*-/-*^ mice with *cps1-1*, an avirulent *Toxoplasma* uracil auxotroph, results in protective immunity to lethal challenge [33]. In parallel, we discovered that the *Toxoplasma* Type I strain RH triggers an unusual autophosphorylation pathway of p38 mitogen-activated protein kinase (MAPK) activation, and that this response drives IL-12 production in mouse bone marrow-derived macrophages [34]. This finding was later extended by others who identified the parasite dense granule secretory protein GRA24 as the activator of p38 MAPK [35,36]. Thus, GRA24 is targeted by the parasite into the host cell where it binds and triggers allosteric autoactivation of p38 MAPK. This results in nuclear translocation and changes in host gene transcription, including upregulation of genes controlling IL-12 transcription.

In the present study, we investigate the role of GRA24 in MyD88-independent triggering of immunity both *in vitro* and *in vivo*. Using genetically engineered *cps1-1* GRA24 KO parasites, we show that this molecule drives p38 phosphorylation and IL-12 production independently of MyD88 signaling. In addition, we identify a novel subset of cytokines and chemokines that are controlled by GRA24 during *in vivo* infection. Furthermore, we show that GRA24 triggers a strong host defense response during induction of immunity by *cps1-1*. Our results provide a striking molecular example illustrating the evolutionary adaptation of *Toxoplasma* to actively trigger inflammatory cytokine production to promote host survival, parasite latency, and transmission.

## Results

### *Toxoplasma* triggers MyD88-independent IL-12 production during *in vivo* and *in vitro* infection

**Fig 1:**
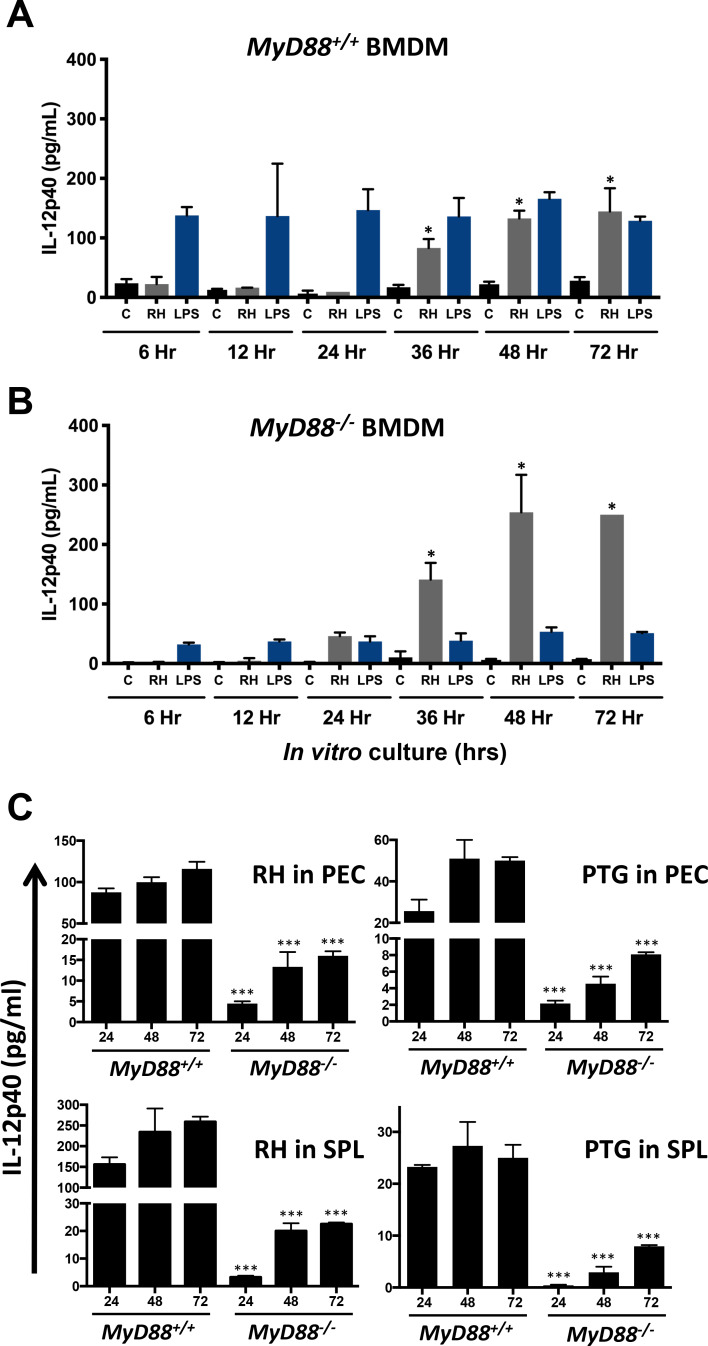
*Toxoplasma gondii* triggers production of MyD88-independent IL-12 during *in vivo* and *in vitro* infection. Bone marrow-derived macrophages (BMDM) from wild-type (**A**, *MyD88*^*+/+*^) and MyD88 knockout (**B**, *MyD88*^*-/-*^) mice were cultured in medium (C), infected with RH strain tachyzoites (1:1 ratio of parasite to cells) or stimulated with LPS (100 ng/ml). At the indicated time points, supernatants were collected for cytokine ELISA. (**C**) *MyD88*^*+/+*^ and *MyD88*^*-/-*^ mice (n = 2 per group) were infected by intraperitoneal inoculation with 10^3^ RH or PTG strain tachyzoites. Four days later, peritoneal exudate cells (PEC) and splenocytes (SPL) were isolated and cultured with no further stimulation. Supernatants were collected for cytokine ELISA at the indicated time points (hr). Data shown are the means ± SD of cells cultured in triplicate. Statistical significance was assessed using Student’s t test (* p < 0.05, *** p < 0.001). These experiments were performed three times with the similar result.

To understand the MyD88-independent IL-12 response during *T*. *gondii* infection, we generated bone marrow-derived macrophages (BMDM) from *MyD88*^*+/+*^ and *MyD88*^*-/-*^ mice then stimulated the cells with Type I RH tachyzoites and lipopolysaccharide (LPS). We found increasing amounts of IL-12p40 production as infection progressed in *MyD88*^*+/+*^ (Fig 1A) and *MyD88*^*-/-*^ (Fig 1B) BMDM infected with RH compared to media control. Nevertheless, we note that the cytokine was not produced at detectable levels until 24–36 hr after infection. In contrast, the response to LPS, which signals through TLR4 and MyD88, was largely abrogated in MyD88 KO BMDM, and in WT cells the maximum response was achieved within 6 hr of stimulation (Fig 1A and 1B). The parasites initiate egress at approximately 48 hr post-infection, likely accounting for lack of IL-12 increase between 48 and 72 hr. Although IL-12p40 levels were somewhat higher in *MyD88*^*-/-*^ BMDM compared to *MyD88*^*+/+*^ BMDM infected with RH in this experiment, over biological replicates (n = 3) this result was not consistent. We examined BMDM IL-12p40 production following infection with Type II strain tachyzoites that have been reported to induce a more robust IL-12 response compared to Type I strains [37]. While there were some minor differences, we found overall that IL-12p40 production was very similar following infection with RH and Type II PTG in both *MyD88*^*+/+*^ (S1A Fig) and *MyD88*^*-/-*^ (S1B Fig) BMDM. Caspase-8 has recently been shown to contribute to IL-12 production and antimicrobial defense to *Toxoplasma* independent of RIPK3 [38]. Therefore, we wanted to determine if RIPK3-independent caspase-8 signaling contributed to parasite-induced IL-12 in BMDM. We infected *RIPK3*^*-/-*^ and *RIPK3*^*-/-*^*Caspase8*^*-/-*^ BMDM with either RH or PTG tachyzoites and measured IL-12p40 in the supernatants. The results showed *RIPK3*^*-/-*^ and *RIPK3*^*-/-*^*Caspase8*^*-/-*^ BMDM infected with either RH or PTG produced similar amounts of IL-12p40 production compared to WT BMDM (S2A and S2B Fig). We conclude that caspase-8 is not involved in the response to *T*. *gondii* triggered in BMDM.

We also assessed IL-12p40 production in peritoneal exudate cells (PEC) and splenocytes (SPL) in *MyD88*^*+/+*^ and *MyD88*^*-/-*^ mice that were infected by intraperitoneal (i. p.) inoculation with either Type I RH or Type II PTG tachyzoites. There was a strong IL-12p40 response in the *MyD88*^*+/+*^ PEC and SPL infected with both RH and PTG parasite strains (Fig 1C). The IL-12 response was less robust in PEC and SPL from infected MyD88 KO mice. Nevertheless, there was clear IL-12 production elicited by both parasite strains independent of MyD88 (Fig 1C). Together, our results identify an alternative MyD88-independent pathway of IL-12p40 production triggered by Type I and Type II strains of *Toxoplasma* during both *in vitro* and *in vivo* infection.

### Dendritic cells, neutrophils and macrophages are sources of IL-12 production in *T*. *gondii* infected MyD88 deficient mice

**Fig 2:**
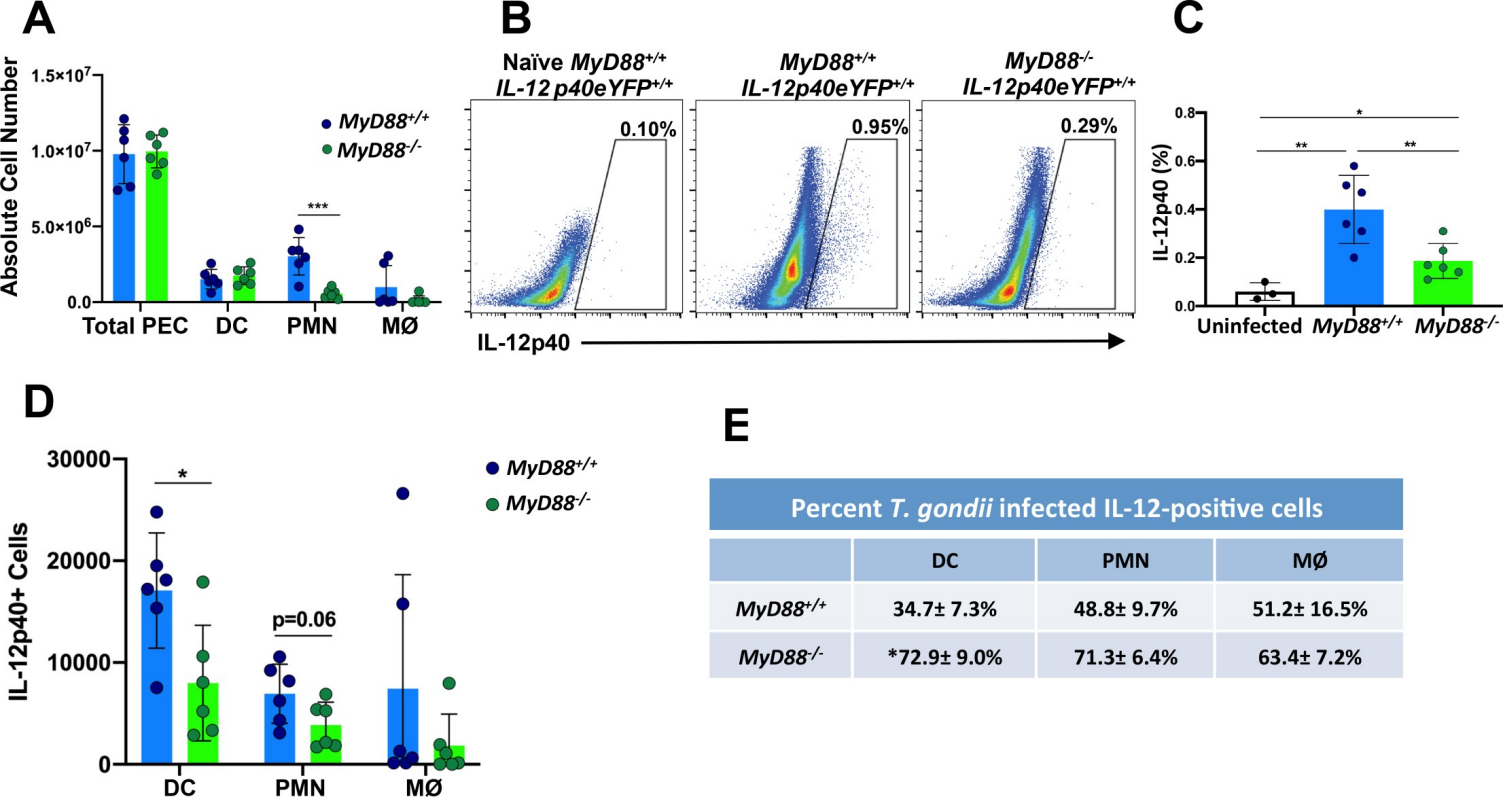
Multiple cell types produce MyD88-independent IL-12 during *in vivo* infection. IL-12 reporter mice on a wild type (*MyD88*^*+/+*^*IL-12p40*^*eYFP/eYFP*^) and MyD88 knockout (*MyD88*^*-/-*^*IL-12p40*^*eYFP/eYFP*^) background were infected by intraperitoneal injection with 10^3^ RH strain tachyzoites. On day 5 post-inoculation, peritoneal exudate cells were collected for *ex vivo* analysis. (**A**) Number of PEC, dendritic cells (DC), neutrophils (PMN) and macrophages (MØ) present in the peritoneal cavity. (**B**) Percentage of IL-12eYFP positive cells in the peritoneal cavity of one representative infected *MyD88*^*+/+*^*IL-12p40*^*eYFP/eYFP*^, one representative infected *MyD88*^*-/-*^*IL-12p40*^*eYFP/*^*eYFP*, and one representative noninfected *MyD88*^*+/+*^*IL-12p40*^*eYFP/eYFP*^ mouse. (**C**) Percent IL-12 positive cells in the peritoneal cavities of WT and MyD88 KO IL-12 reporter mice. (**D**) Number of IL-12 positive cells amongst DC, PMN and MØ isolated from *MyD88*^*+/+*^ and *MyD88*^*-/-*^ reporter mice. (**E**) Percent of infected cells that are positive for IL-12. In these experiments, DC were defined as MHCII^+^, CD11c^+^, F4/80^-^ cells; PMN were defined as Ly6G^+^ cells; MØ were defined as F4/80^+^ cells. Values are the means ± SEM of 2 pooled independent experiments, n = 6 per group. Each symbol represents an individual mouse. Statistical significance was assessed using Student's t test (* p < 0.05, ** p < 0.01, *** p < 0.001) (**A**, **C** and **D**). Mann-Whitney test was used to determine statistical significance of IL-12-positive MyD88 WT vs. KO DC in **E**. These experiments were performed three times with similar results.

To understand the cells involved in IL-12 production in MyD88 deficient mice during *T*. *gondii* infection, we utilized IL-12eYFP reporter mice, a bicistronic reporter strain in which cells expressing IL-12 also express eYFP [39]. We crossed *MyD88*^*+/+*^*IL-12p40*^*eYFP/eYFP*^ with *MyD88*^*-/-*^ animals to generate strains expressing IL-12eYFP on *MyD88*^*+/+*^ and *MyD88*^*-/-*^ backgrounds. The reporter mouse strains were infected with RH tachyzoites by i. p. inoculation and we then used flow cytometry to measure percentages and total cell numbers in the peritoneal cavity five days post infection. Our data show similar numbers of cells in the peritoneal cavity of *MyD88*^*+/+*^*IL-12p40*^*eYFP/eYFP*^ and *MyD88*^*-/-*^*IL-12p40*^*eYFP/eYFP*^ infected mice at this time point (Fig 2A). There were also similar numbers of DC in both mouse strains, but there was a 3–5 fold decrease in neutrophils (PMN) and macrophages (MØ) in the absence of MyD88 (Fig 2A). In line with the data shown in Fig 1C, IL-12-positive cells in *MyD88*^*-/-*^ mice were present at approximately 30% of the levels in the IL-12 reporter *MyD88*^*+/+*^ animals (Fig 2B and 2C). In Fig 2D we examined the relative contributions of DC, PMN and MØ to the IL-12-positive populations in *MyD88*^*+/+*^ and *MyD88*^*-/-*^ mice. While each cell type contributed to the IL-12-positive population, DC contributed the largest portion in the WT strain. In contrast, the proportions were more evenly divided in the KO mice (Fig 2D). By staining for intracellular tachyzoites we determined the percent infection in each of the IL-12-positive populations (Fig 2E). In the *MyD88*^*+/+*^ reporter strain, most of the IL-12-positive DC, PMN and MØ were uninfected. Interestingly, the majority of IL-12-positive cells were infected on the *MyD88*^*-/-*^ background, regardless of identity (Fig 2E). Collectively, these data show that the cell types producing IL-12p40 in both MyD88 sufficient and MyD88 deficient mice are dendritic cells, neutrophils and macrophages. In addition, the MyD88-independent IL-12 that is produced derives predominantly from infected cells.

### Parasite GRA24 plays a key role in MyD88-independent p38 MAPK-dependent IL-12 production

**Fig 3:**
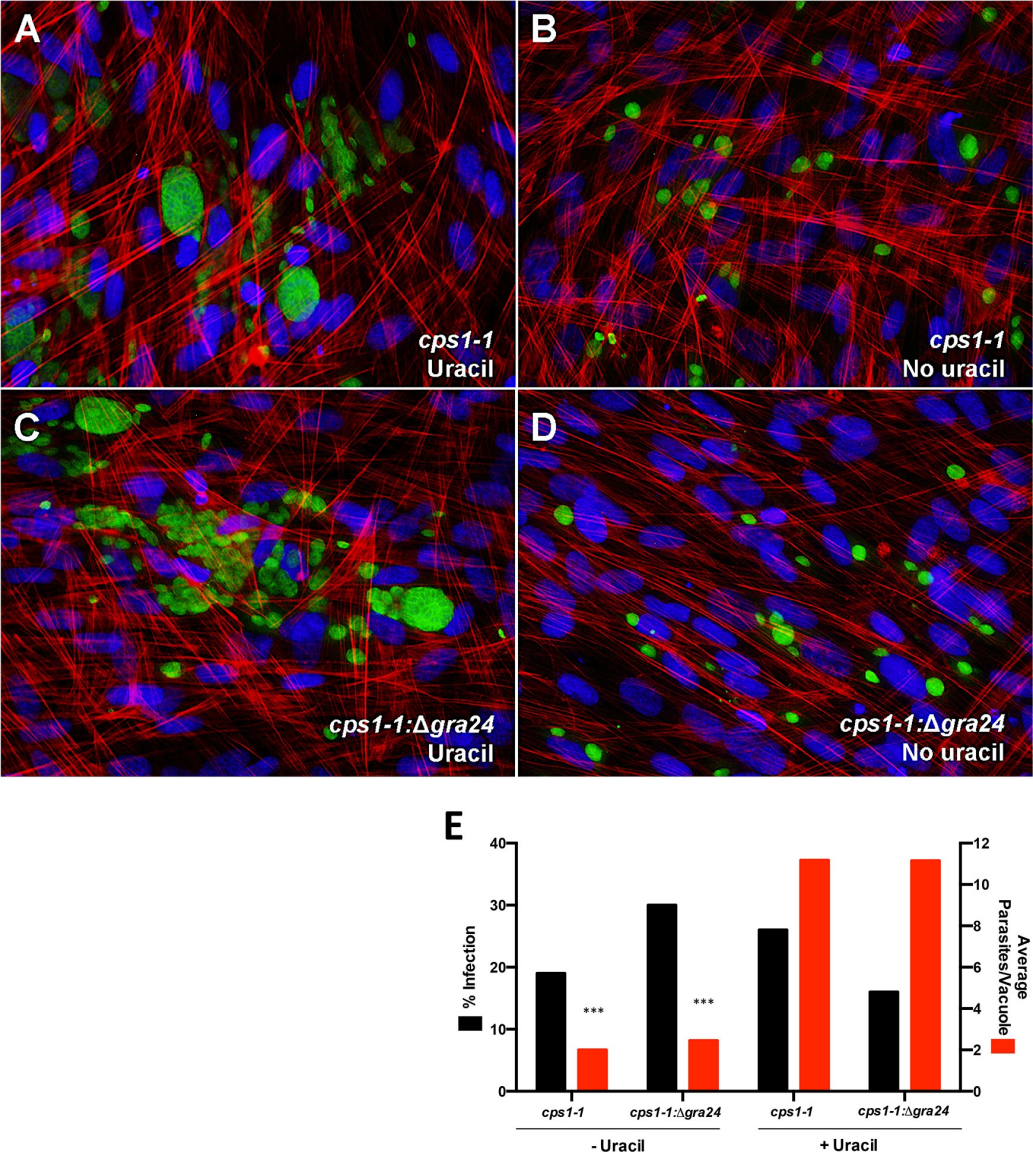
Both *cps1-1* and *cps1-1*:*Δgra24* tachyzoites require supplemental uracil for sustained replication. Tachyzoites of strains *cps1-1* or *cps1-1*:*Δgra24* were inoculated onto human foreskin fibroblast monolayers in the presence (**A** and **C**) or absence (**B** and **D**) of exogenous uracil. Two days after infection, monolayers were fixed and stained with FITC conjugated anti-*Toxoplasma* antibody (green), phalloidin-Texas red to stain host cell actin, and DAPI (blue) to stain nuclei. Images were collected under 40x magnification. (**E)** Percent infection and the average parasites per vacuole were quantified in *cps1-1* and *cps1-1*:*Δgra24* infected fibroblasts in the presence and absence of exogenous uracil. This experiment was repeated twice with the same result. Data shown are the mean counts of three independent experiments. Statistical significance was assessed using Mann-Whitney test comparing *cps1-1* (+/-) exogenous uracil and *cps1-1*:*Δgra24* (+/-) exogenous uracil (**E**) (*** p < 0.001).

*T*. *gondii* dense granule protein GRA24 has been shown to trigger p38 MAPK-dependent IL-12 [35]. To examine the contribution of GRA24 to MyD88-independent IL-12, we assessed IL-12 production by the uracil auxotroph strain Δ*ompdc*Δ*up* (designated as *cps1-1*) and the corresponding GRA24 knockout strain Δ*ompdcup*Δ*gra24* (designated as *cps1-1*: Δ*gra24*) [40,41]. Inclusion or exclusion of exogenous uracil enabled us to determine the influence of tachyzoite proliferation on IL-12 production and MAPK activation. Fig 3 shows images of *cps1-1* (panel A) and *cps1-1*:*Δgra24* (panel C) on fibroblasts in the presence of uracil. At this time point (48 hr post-infection), cells were heavily infected and parasite egress was readily observed with uracil supplementation. In parallel cultures without uracil (Fig 3B and 3D), growth of both strains was restricted to ≤ 2 parasite divisions. Percent infection and average parasites per vacuole were measured in the *cps1-1* and *cps1-1*:*Δgra24* infected fibroblasts in the presence and absence of exogenous uracil (Fig 3E). We found that presence or absence of GRA24 had no significant effect on infection or replication rate whether or not exogenous uracil was provided.

**Fig 4:**
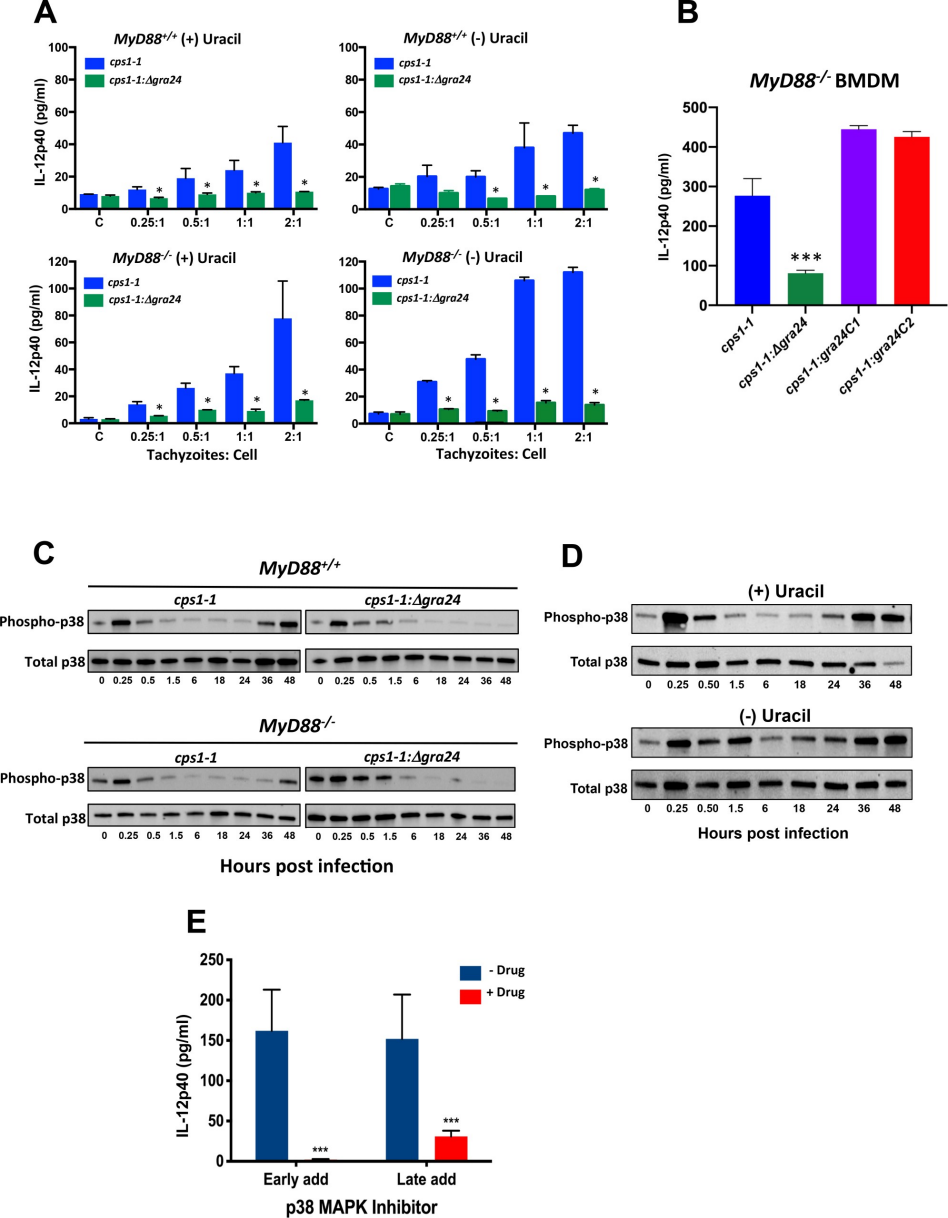
GRA24 controls MyD88-independent p38 MAPK-dependent IL-12 production. (**A**) *MyD88*^*+/+*^ and *MyD88*^*-/-*^ BMDM were infected with *cps1-1* or *cps1-1*:*Δgra24* tachyzoites at the indicated parasite to cell ratios. Cultures were initiated with (+) and without (-) exogenous uracil and supernatants were collected 48 hr after infection. C, control non-infected cells. (**B**) *MyD88*^*-/-*^ BMDM were inoculated with *cps1-1*, *cps1-1*:*Δgra24*, *cps1-1*:*gra24C1* or *cps1-1*:*gra24C2* tachyzoites at a 2:1 parasite to cell ratio. Supernatants were collected 48 hr post infection for IL-12 measurement. Production of IL-12 was significantly reduced in cells infected with *cps1-1*:*Δgra24* in pairwise comparisons with *cps1-1* and the two complementation mutants. (**C**) *cps1-1* or *cps1-1*:*Δgra24* tachyzoites were inoculated onto *MyD88*^*+/+*^ and *MyD88*^*-/-*^ BMDM at a ratio of 2:1. Cultures were briefly centrifuged to initiate parasite and cell contact, then lysates were prepared for Western blot assay at the indicated time points after infection (hr). (**D**) WT BMDM were infected with *cps1-1* parasites in the presence and absence of uracil, then lysates were prepared for Western blot analysis as in (**C**). (**E**) WT BMDM were infected with *cps1-1* tachyzoites in the presence and absence of p38 MAPK inhibitor SB202190 (10 μM). Supernatants were collected 36 hr after infection for cytokine ELISA. The inhibitor was added either 1 hr prior to infection (“Early add”) or 10 hr after infection (“Late add”). Data shown are the means ± SD of cells cultured in triplicate. Statistical significance was evaluated using Student’s t test (* p < 0.05, *** p < 0.001). These collective experiments were repeated with the similar result two times.

*MyD88*^*+/+*^ and *MyD88*^*-/-*^ BMDM were inoculated with either *cps1-1* and *cps1-1*:*Δgra24* tachyzoites and supernatants were collected 48 hr later for cytokine assay (Fig 4A). In the presence of exogenous uracil, proliferating *cps1-1* parasites triggered IL-12p40 in both WT and KO BMDM (Fig 4A, left panels). Strikingly, these responses failed to occur using GRA24-negative parasites. Because parasite-induced IL-12 was not produced until 24–36 hr post-infection, it was possible the response was tied to tachyzoite proliferation. Accordingly, the experiment was conducted in the absence of exogenous uracil. Even when proliferation was restricted, *cps1-1* tachyzoites maintained the ability to induce IL-12 in dependence upon GRA24 (Fig 4A, right panels). We also generated two independent GRA24 complementation mutants, designated *cps1-1*:*gra24C1* and *cps1-1*:gra24C2, and measured IL-12p40 production in BMDM. As expected, restoration of GRA24 expression in *cps1-1*:*Δgra24* parasites also reestablished production of IL-12 (Fig 4B).

We next examined the influence of GRA24 on p38 MAPK activation in the context of *cps1-1* infection of BMDM. Interestingly, infection triggered an early wave of p38 phosphorylation (≤ 15 min post infection) followed by dephosphorylation and a later wave that occurred 36 hr after infection (Fig 4C). Both responses occurred independently of MyD88, but the second wave was strictly dependent upon GRA24 (Fig 4C). Type I *Toxoplasma* is known to activate both STAT3 and ERK1/2 [42–44]. Accordingly, we examined the influence of MyD88 and GRA24 on these responses. We found neither host MyD88 nor parasite GRA24 influenced ERK1/2 phosphorylation (S3A Fig) or STAT3 phosphorylation (S3B Fig). The experiment in Fig 4C was carried out in the presence of uracil, and we therefore wondered if the second wave of p38 activation was driven by parasite proliferation and possibly host cell lysis and reinfection of new cells. Accordingly, we examined *cps1-1*-driven p38 MAPK activation under proliferation permissive (+ uracil) and proliferation non-permissive (- uracil) conditions (Fig 4D). Even when parasite replication was prevented by exclusion of uracil, we saw the same two-wave pattern of p38 MAPK phosphorylation.

Production of GRA24-dependent IL-12 correlated most closely with the late wave of p38 phosphorylation, which was also dependent upon GRA24. To formally demonstrate that late p38 phosphorylation was the key event in *cps1-1*-induced IL-12 production, we employed timed addition of the small molecule p38 MAPK inhibitor SB202190 [45]. Addition of the inhibitor 1 hr prior to infection (“Early add”) completely prevented IL-12 production during infection (Fig 4E), as measured in 48 hr supernatants. Addition of the inhibitor 10 hr after infection (“Late add”) also almost completely blocked the IL-12 response. Thus, the second wave of p38 activation stimulated by GRA24 underlies delayed IL-12 production in *Toxoplasma* infected BMDM.

### GRA24 regulates IL-12p40 and other cytokines and chemokines during *in vivo* infection

**Fig 5:**
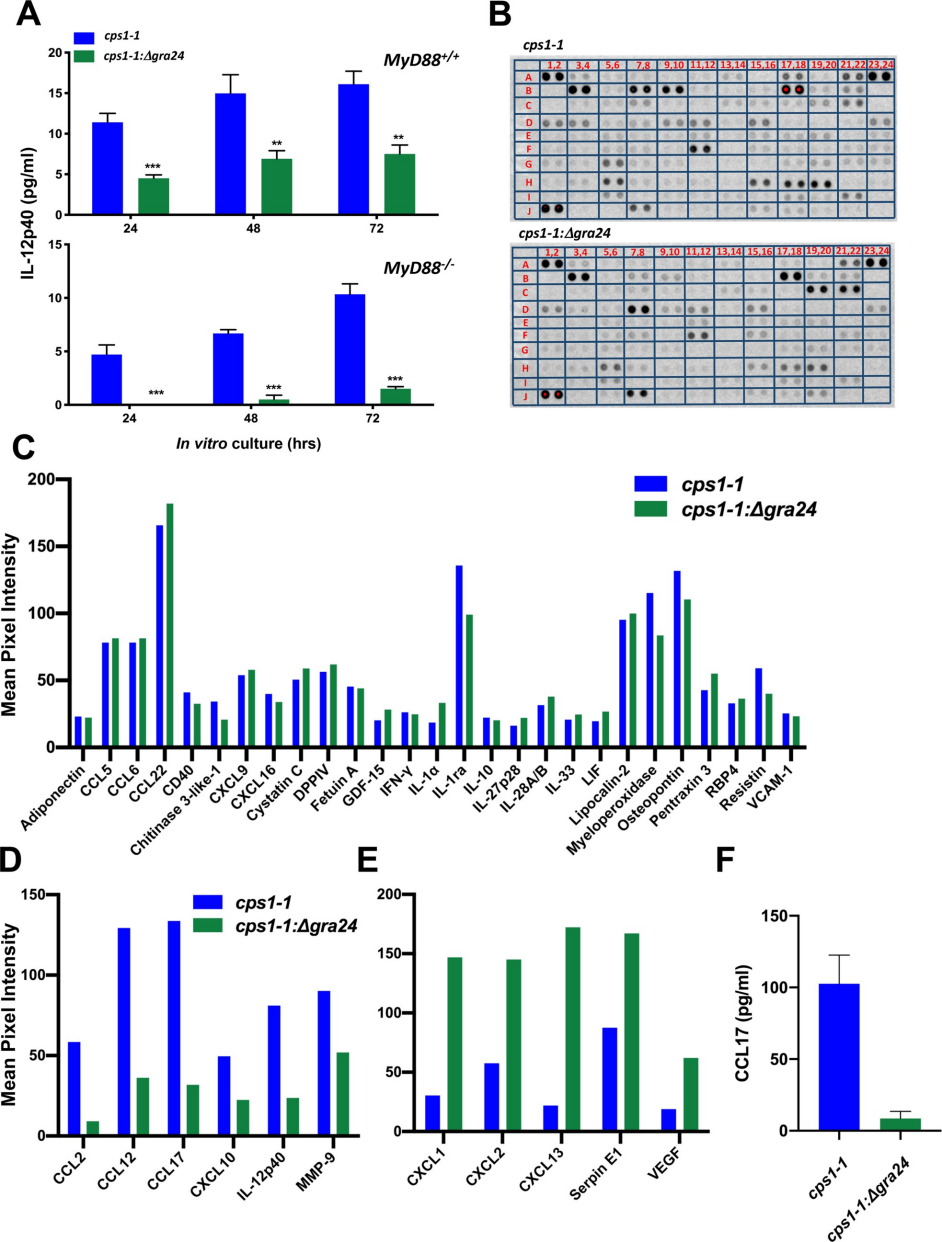
GRA24 controls the up-regulation of IL-12p40 and other cytokines and chemokines during *T*. *gondii* infection. (**A**) *MyD88*^*+/+*^ and *MyD88*^*-/-*^ mice (n = 3 per group) were infected by intraperitoneal injection of 10^6^ *cps1-1* or *cps1-1*:*Δgra24* tachyzoites. Four days later, cells were harvested from the peritoneal cavity and cultured without further stimulation for the indicated times. (**B**) *MyD88*^*+/+*^ mice (n = 2 per group) were infected by i. p. inoculation with 10^6^ *cps1-1* or *cps1-1*:*Δgra24* tachyzoites. Four days later, peritoneal exudate cells were harvested and cultured for 72 hr. The supernatants were harvested and prepared for cytokine proteome array. (**C**) Cytokine mean pixel intensity of the proteomic cytokine array in (**B**) that were equivalent between *MyD88*^*+/+*^ mice infected with *cps1-1* and *cps1-1*:*Δgra24*, and that were greater than 20 fold up-regulated above background. (**D**) Cytokines up-regulated in *MyD88*^*+/+*^ mice infected with *cps1-1* relative to *cps1-1*:*Δgra24* in the cytokine proteome array. (**E**) Cytokines in the proteomic array that were up-regulated in *cps1-1*:*Δgra24* relative to *cps1-1* in infected *MyD88*^*+/+*^ mice. (**F**) *MyD88*^*+/+*^ mice (n = 2 per group) were infected, cells were collected and cultured as in (**B**). The supernatants were harvested and CCL17 was measured by ELISA. Data shown are the means ± SD of cells cultured in triplicate. Statistical significance was assessed using Student’s t test (** p < 0.001, *** p < 0.001). These collective experiments were repeated 2–3 times.

To determine if GRA24 controls production of IL-12p40 during *in vivo* infection, *MyD88*^*+/+*^ and *MyD88*^*-/-*^ mice were infected with either *cps1-1* or *cps1-1*:*Δgra24* by i. p. injection. Four days post infection, PEC were harvested and cultured without further stimulation. IL-12p40 was measured in the supernatants at 24 hr, 48 hr and 72 hr. In line with the BMDM responses, *cps1-1* infected *MyD88*^*+/+*^ PEC produced approximately twice as much IL-12p40 compared to the *cps1-1*:*Δgra24* infected mice (Fig 5A). Furthermore, there was a near complete ablation of IL-12 production in MyD88 KO mice infected with the GRA24 deletion mutant (Fig 5A). To determine if GRA24 controls expression of other chemokines and cytokines, we used a proteomic array to screen a total of 111 cytokines, chemokines and related immune mediators released by PEC isolated from WT mice infected with *cps1-1* and *cps1-1*:*Δgra24*. Fig 5B shows that overall, a large collection of cytokines and chemokines were up-regulated in PEC isolated from mice infected with each parasite strain. A schematic showing the coordinates of each cytokine and chemokine on the proteomic array is shown in S4 Fig. A collection of immune-related factors were up-regulated similarly during infection with the two parasite strains. Fig 5C shows factors up-regulated 20-fold or greater above background by both parasite strains. A subset of cytokines and chemokines were clearly up-regulated in dependence upon GRA24, including IL-12p40 and CCL17 (Fig 5D). Interestingly, we also identified a subset of immune-related mediators whose expression was negatively regulated by GRA24 including CXCL1 and CXCL2 (Fig 5E). As independent confirmation of these results, we used ELISA to measure CCL17. The result shows CCL17 production is clearly dependent upon GRA24 (Fig 5F). Together, these results demonstrate that GRA24 positively and negatively regulates distinct sets of cytokines and chemokines during *in vivo T*. *gondii* infection.

### GRA24 plays a role in protective immunity independent of the MyD88 adaptor protein

**Fig 6:**
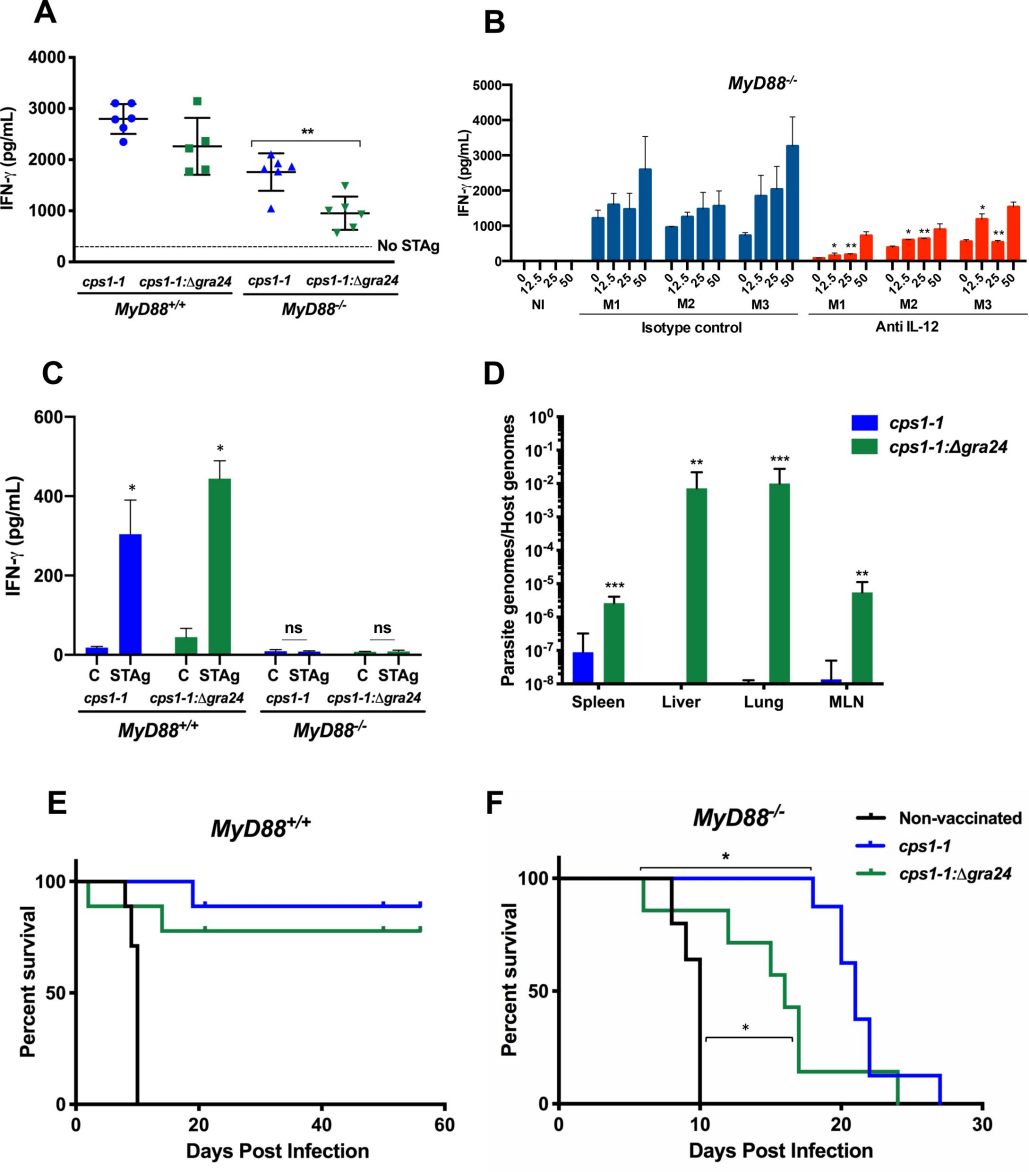
GRA24 induces protective immunity in the absence of MyD88. (**A**) *MyD88*^*+/+*^ and *MyD88*^*-/-*^ animals (n = 5–6 mice per group) were i. p. inoculated at Day 0 and Day 14 with 10^6^ *cps1-1* or *cps1-1*:*Δgra24* tachyzoites, then at day 28 splenocytes were isolated and subjected to *in vitro* stimulation with soluble tachyzoite antigen (STAg; 50 μg/ml). Supernatants were collected at 72 hr for cytokine ELISA. Each point represents a single mouse. (**B**) *MyD88*^*-/-*^ mice (n = 3 mice per group) were administered IL-12 neutralizing antibody or isotype control antibody during the course of infection initiated by inoculation with 5 x 10^6^ *cps1-1* tachyzoites. At Day 10 post-infection, splenocytes were isolated and stimulated with STAg at the indicated concentrations (μg/ml), then 72 hr later supernatants were collected for IFN-γ assay. NI, splenocytes from noninfected mice. M1, M2, M3 are individual *MyD88*^*-/-*^ mice treated with the isotype control and M4, M5, M6 are individual *MyD88*^*-/-*^ mice treated with the IL-12 neutralizing antibody. (**C**) Mesenteric lymph node cells were pooled from vaccinated mice, and subjected to STAg re-stimulation and cytokine assay as in (**A**). C, control cells cultured in medium alone. (**D**) *MyD88*^*-/-*^*IL-12p40*^*eYFP/eYFP*^ mice (n = 7 per group) were vaccinated by i. p. inoculation with *cps1-1* or *cps1-1*:*Δgra24* tachyzoites. Two weeks after the final inoculation, mice were challenged with 10^3^ RH tachyzoites. Nine days post challenge tissues were harvested from each individual mouse and processed independently for parasite quantitation by qPCR. In **E** and **F**, *MyD88*^*+/+*^ and *MyD88*^*-/-*^ animals were vaccinated by i. p. inoculation with *cps1-1* or *cps1-1*:*Δgra24* tachyzoites as in (**D**). Two weeks after the final inoculation, mice were challenged by subcutaneous injection with 10^3^ RH strain tachyzoites, and survival was monitored. Data shown are the means ± SD of cells cultured in triplicate (**A**, **B** and **C**). Statistical significance was evaluated using Student’s t test (**A**, **C**, **D**). A Mann-Whitney test was used to measure statistical significance between isotype controls and the corresponding anti-IL-12 concentrations (**B**). Log rank Mantel-Cox test was used to measure the statistical significance between the survival curves. (**E** and F). The asterisks in **F** indicate *MyD88*^*-/-*^ mice vaccinated with the *cps1-1*:*Δgra24* strain were significantly more susceptible than *cps1-1* vaccinated mice, and that animals vaccinated with the *cps1-1*:*Δgra24* strain were significantly more resistant than the nonvaccinated group. For the results shown, * p < 0.05, ** p < 0.01, and *** p < 0.001. These collective experiments were repeated with the same result two to three times.

Vaccination with *cps1-1* induces a strong Th1 response and protective immunity in both *MyD88*^*+/+*^ and *MyD88*^*-/-*^ mice [33]. We wanted to determine the role of GRA24 in the response. Accordingly, we employed a vaccination protocol involving two sequential inoculations with *cps1-1* and *cps1-1*:*Δgra24* parasite strains. Two weeks after the final inoculation, a splenocyte cytokine recall assay was performed using soluble tachyzoite antigen (STAg). The results (Fig 6A) revealed that vaccination of WT mice with either *cps1-1* or *cps1-1*:*Δgra24* tachyzoites induced a strong IFN-γ response. However, the splenic recall response in *cps1-1*:*Δgra24* vaccinated MyD88 KO mice was significantly weaker than the corresponding *cps1-1* response (Fig 6A). Production of IFN-γ during *T*. *gondii* infection is strongly dependent upon IL-12 in wild type mice [46]. To determine if the IFN-γ recall response in *cps1-1* vaccinated *MyD88*^*-/-*^ mice was dependent upon IL-12, we treated *MyD88*^*-/-*^ mice with a depleting anti-IL-12p40 mAb or rat immunoglobulin isotype control over the course of an i. p. infection. One week following infection, splenocytes were subjected to an *in vitro* recall assay with STAg. The results (Fig 6B) show that *in vivo* depletion of IL-12 substantially reduces the IFN-γ response relative to the Ab control-treated mice. We also examined the IFN-γ response in mesenteric lymph nodes in the vaccinated mice. Interestingly, production of IFN-γ was completely dependent upon MyD88 at this location (Fig 6C).

To determine if GRA24 was responsible for protective immunity elicited by *cps1-1*, we determined how *cps1-1* vaccination with or without GRA24 impacted systemic parasite burden in MyD88 deficient mice following challenge infection with virulent RH strain tachyzoites. Nine days after challenge, spleen, liver, lungs and MLN were collected and parasite burden was measured by quantitative PCR measurement of the *Toxoplasma* B1 gene relative to the host argininosuccinate lyase gene. As shown in Fig 6D, there was a dramatic increase in parasite burden in all four organs in the *cps1-1*:*Δgra24* vaccinated mice compared to the *cps1-1* vaccinated mice.

To further elucidate the impact of GRA24 during vaccination in induction of protective immunity, we monitored survival in lethally challenged animals. Non-vaccinated *MyD88*^*+/+*^ and *MyD88*^*-/-*^ mice died by day 10 after challenge (Fig 6E and 6F). In contrast, *MyD88*^*+/+*^ mice vaccinated with *cps1-1* and *cps1-1*:*Δgra24* were resistant to challenge (Fig 6E). While WT mice vaccinated with *cps1-1*:*Δgra24* appeared slightly less resistant, this result was not statistically significant. *MyD88*^*-/-*^ mice vaccinated with *cps1-1* were resistant to RH inoculation, with animals beginning to succumb at approximately 3 weeks post-challenge. In striking contrast MyD88 KO animals vaccinated with the GRA24 deletion mutant were significantly more susceptible to challenge (Fig 6F). We also note that this group of mice appeared more resistant than non-vaccinated counterparts, indicating the presence of protective mechanisms that involve neither GRA24 nor MyD88. Regardless, these collective results demonstrate that GRA24 elicits MyD88 independent immunity, thereby limiting systemic dissemination and enhancing host survival.

## Discussion

The TLR/MyD88 signaling module in mice underlies resistance to a large number of pathogens, including *T*. *gondii*. However, the emerging realization that this axis of immunity is likely less critical for anti-microbial defense in humans has prompted significant interest in identifying MyD88-independent pathways of resistance. Here, we identify an MyD88-independent pathway of immunity to *T*. *gondii* that relies upon host p38 MAPK phosphorylation initiated by parasite dense granule protein GRA24. Activation of p38 MAPK by GRA24 triggers production of IL-12 during *in vitro* and *in vivo* models of infection. We also identified a novel subset of cytokines and chemokines whose expression during *in vivo* infection was upregulated by GRA24, and a unique subset that was down-regulated by this dense granule protein. In a vaccination model employing the uracil auxotrophic *Toxoplasma* strain *cps1-1*, we found that GRA24 drives an MyD88-independent Type I cytokine response associated with production of IFN-γ and protection from lethal challenge infection.

Evidence for an unusual pathway of *T*. *gondii*-driven p38 MAPK autophosphorylation during *in vitro* infection of mouse macrophages was first uncovered several years ago [34]. GRA24 was subsequently identified as the critical parasite effector protein using in silico methods to predict proteins capable of both entering the parasite secretory pathway and targeting the host cell nucleus [35,47,48]. GRA24 is unusual in that it has no homology to genes in other apicomplexa. Interestingly, the closely related apicomplexan *Neospora caninum* uses a distinct pathway to phosphorylate host p38 MAPK dependent upon G-protein-coupled receptor signaling [49]. In contrast to our results, p38 MAPK activation by *N*. *caninum* is associated with IL-12 down-regulation and evasion of host immunity.

Like other dense granule proteins, GRA24 is composed of intrinsically disordered regions that are predicted to favor its activity as a host-directed effector molecule [50]. The protein is exported across the parasitophorous vacuole membrane using a translocation pathway involving parasite proteins MYR1 and ASP5 [51–53]. GRA24 subsequently binds to p38α MAPK, triggering allosteric autoactivation, nuclear translocation and upregulation of transcription factors such as Egr-1 and c-fos [35,54]. Despite a relatively clear picture of GRA24 function inside the host cell, our understanding of the importance of this parasite molecule in MyD88-independent pathways of Th1-induction and protective immunity has been relatively limited.

Our *in vitro* infection experiments revealed a bi-phasic pattern of p38 MAPK activation in which the kinase was activated in a GRA24-independent manner within minutes of infection, followed by a second GRA24-dependent activation occurring 24–36 hr later. The second wave occurred even when replication and egress was prevented by excluding exogenous uracil, a result that argues against cell lysis and secondary infection as the stimulus for GRA24-dependent p38 MAPK phosphorylation. While we do not understand the underlying basis for this pattern, it may be noteworthy that GRA24 appears in the host cell nucleus only at later points in infection [35]. Regardless, it appears that the delayed wave of GRA24-dependent p38 MAPK phosphorylation is the critical driver of IL-12. First, IL-12 is only produced at later time points (36–48 hr) of BMDM infection, in contrast to LPS that triggers a response within the first 6 hr of stimulation. Second, addition of a pharmacological p38 MAPK inhibitor during the eclipse phase of parasite-induced p38 phosphorylation (10 hr post-infection) potently blocked subsequent IL-12 secretion.

The cause and significance of the early MyD88-independent p38 MAPK activation event is unclear at present. However, it is interesting to note that *Toxoplasma*-triggered STAT3 and STAT6 activation follows a similar pattern, with an early ROP16-independent phase followed by a later phase dependent upon this parasite kinase [42]. In this case, early STAT3 activation is dependent upon a FAK-Src-STAT3 signaling pathway associated with invasion [55]. We are currently examining whether this pathway also triggers early p38 MAPK activation.

We were able to induce an MyD88-independent protective immune response that depended upon GRA24 activity in a vaccine strain of the parasite. Yet, we also observed that even in the absence of this dense granule protein there remained a significant MyD88-independent protective response. The latter result provides strong evidence for the existence of other pathways of protection that rely on neither host TLR/MyD88 nor parasite GRA24. A recent study employing human cells revealed that peripheral blood monocytes and dendritic cells produce IL-12 only after phagocytosis of live tachyzoites, a process not generally associated with involvement of TLR or MyD88 [56]. It has also been shown that TLR11-independent inflammasome activation triggers Th1 immunity during *Toxoplasma* infection and the neutrophils can provide an early source on IFN-γ independently of TLR signaling [57,58]. It is possible that these phenomena contribute to MyD88-independent activation of immunity in the mouse model employed here. It has also been reported that the *T*. *gondii* dense granule protein GRA15 possesses proinflammatory cytokine-inducing activity. GRA15 is trafficked to the parasitophorous vacuole membrane where it activates NFκB signaling independently of MyD88. This in turn leads to induction of IL-12 [59]. However, GRA15 from Type I parasite strains (including the *cps1-1* strain used here) does not display this activity and is therefore unlikely to account for *cps1-1*-induced, GRA24-independent immunity in *MyD88*^*-/-*^ mice.

Dense granule proteins GRA24 and GRA15 are members of a growing family of *Toxoplasma* host-directed effectors that target signaling to remodel cell function [60]. Parasite GRA6 activates host transcription factor NFAT4 through binding to calcineurin activator calcium-modulating ligand (CAMLG) [61]. GRA6-dependent NFAT4 activation results in production of the chemokines CCL2 and CXCL2 which together promote recruitment of inflammatory monocytes and neutrophils. Because these cells are targets of infection, they may play a role in parasite dissemination. By interacting with herpesvirus-associated ubiquitin-specific protease (HAUSP) and the phosphate PP2A-B55, GRA16 alters steady-state levels of host p53, which may be important in host cell survival under conditions of stress associated with intracellular infection [47]. Through secretion of dense granule protein TgIST, *Toxoplasma* potently suppresses STAT1-induced gene expression. In the infected cell, this results in generalized nonresponsiveness to the anti-microbial effects of IFN-γ [62,63].

Rhoptries contain host-directed effector molecules that are released early during invasion. Rhoptry protein ROP16 is a host-directed kinase that is injected into the host cytoplasm during invasion where it activates both STAT3 and STAT6 [42,64]. In macrophages, this results in deviation to an M2 phenotype and down-regulation of IL-12 [65]. The *Toxoplasma* rhoptry protein TgWIP is secreted into the host cell where it interacts with the WAVE regulatory complex and SHP2 phosphatase. This impacts actin dynamics in dendritic cells, enhancing host cell motility that in turn promotes parasite dissemination [66]. Together, these studies form the basis for our expanding understanding of *T*. *gondii* as a microbial parasite equipped with a sophisticated toolbox used during infection to deal with the host immune system.

GRA24 is an effector molecule that the parasite deploys to activate signaling leading to IL-12. In one sense this appears counter-intuitive given that this cytokine is critical in activation of immunity leading to parasite elimination [5,46,67]. However, mice lacking IL-12 succumb extremely rapidly to infection before parasite latency is established [11]. Our findings support the model that GRA24 induces MyD88-independent IL-12 production to promote survival of the host in acute infection and parasite survival and transmission by promoting latency. The ability to establish a balance between excessive immunity and inadequate immunity is likely a major factor accounting for the evolutionary success of *Toxoplasma*.

## Materials and methods

### Ethics statement

All experiments performed in this study were conducted in accordance with the Guide for the Care and Use of Laboratory Animals. Protocols were approved by the Institutional Animal Care and Use Committee at the University of New Mexico (Animal Welfare Assurance Number A4023-01). All efforts were made to reduce animal suffering during this study.

### Mice

Female and male mice (6–12 weeks of age) were used in these studies. Female C57BL/6 mice (5 weeks of age) were purchased from Taconic Biosciences Inc. (Rensselaer, NY). Female and male (5 weeks of age) B6.1292(SPJ)-MyD88^tm1.1Defr^/J (*MyD88*^*-/-*^) and B6.129-IL12b^tm1.1Lky^/J (*IL-12p40*^*eYFP/eYFP*^) mouse strains were purchased from The Jackson Laboratory (Bar Harbor, ME). The *MyD88*^*-/-*^ and *IL-12p40*^*eYFP/eYFP*^ mouse strains were crossed in house to generate a *MyD88*^*-/-*^*IL-12p40*^*eYFP*^ strain. The *MyD88*^*-/-*^, *IL-12p40*^*eYFP*^ and *MyD88*^*-/-*^*IL-12p40*^*eYFP*^strains were bred in-house in the University of New Mexico Department of Biology Animal Research Facility. Mouse genotyping was performed by Transnetyx, Inc. (Cordova, TN).

### Parasites

Confluent human foreskin fibroblast monolayers (HFF; ATCC, Manassas, VA) were infected with *T*. *gondii* Type I (RH) or Type II (PTG) strains and passaged weekly into confluent HFF monolayers. HFF were grown to confluence in Dulbecco’s Modified Eagle’s Medium (DMEM) (VWR, Randor, PA) supplemented with 1 mM glutamine and 10% bovine growth serum (FBS; HyClone, Logan, UT), 100 U/ml penicillin (ThermoFisher Scientific, Waltham, MA) and 0.1 mg/ml streptomycin (ThermoFisher Scientific). Construction of the uracil auxotroph strains Δ*ompdc*Δ*up* (designated as *cps1-1*) and Δ*ompdc*Δ*up*Δ*gra24* (designated as *cps1-1*: Δ*gra24*) has previously been described [40,41].

The *cps1-1*: *Δgra24* strain was complemented with a C-terminal HA-tagged copy of GRA24 to independently isolate complemented strains *cps1-1*:*gra24C1* and *cps1-1*:*gra24C2*. The gra24C targeting plasmid was constructed by recombining 5 PCR fragments using forward (F) and reverse (R) primers to synthesize the 5’ target region (F = TTGGGTAACGCCAGGGTTTTCCCAGTCACGACGGTTTAAACCTAGGACAGATG-TCCTCATCTCAGCGTCC; R = GGTGCGACGATTCGCTGCTG), the N-terminal region (F = GGTTGATGAGAGCGGTCCAGC; R = GGGATTATTGTGCCGGGTTGGC), the middle region (F = GGTTCAGCACATGGTAGGCCAAC; R = TCACTCAGGCAC-GCTCTGTATGTC), the C-terminal region (F = TCGGTGTCTCGCTGACATACAGAG; R = GACTTTGTCATCGTCGTCCTTGTAGTCCTCGAGATTACCCTTAGTGGGTGGTTT-AACGATGC), and the 3’ target region (F = CTCGAGGACTACAAGGACGACGATGAC-AAAGTCAAGCTCTACCCATACGATGTTCCAGATTACGCTGCTTAGCCTAGGGTTCAAAGTGCCCTATTGGTACTGGCA; R = GTGAGCGGATAACAATTTCACACAGGAAACA-GCGCGGCCGCACTGCGCTGATACCCGTCGTG) of GRA24. Complemented strains *cps1-1*:*gra24C1* and *cps1-1*:*gra24C2* were isolated after selection in 6-thioxanthine and uracil as previously described [40], and insertion of HA-tagged GRA24 at the GRA24 locus was confirmed using primer pairs (F = CAGCCAACAACGACACTCAGCG; R = GTTGGCCTACCATGTGCTGAACC), and (F = ACCCATACGATGTTCCAGATTACGC; R = CAGGCAACGCCGTGATCCAC).

The mutant parasites were maintained in DMEM (VWR) supplemented with 300 μM uracil, 1% FBS (ThermoFisher Scientific), 100 U/ml penicillin (ThermoFisher Scientific) and 0.1 mg/ml streptomycin (ThermoFisher Scientific). Parasite propagation was maintained by passaging weekly onto a confluent HFF monolayer. To verify that all uracil auxotroph *T*. *gondii* strains maintained dependence upon exogenous uracil, the parasites were passaged into growth medium without supplemental uracil once a month. Parasites were tested for *Mycoplasma* contamination every 3 months employing the MycoProbe detection assay (R & D Systems, Minneapolis, MN).

### Infections and vaccination

*Toxoplasma* was administered to mice by intraperitoneal injection of tachyzoites into mice 6–12 weeks of age. Animals were vaccinated by two intraperitoneal injections (10^6^ tachyzoites) of *cps1-1* or *cps1-1*:*Δgra24* administered two weeks apart. Two weeks after the final vaccination mice were challenged by subcutaneous injection of 10^3^ RH strain tachyzoites.

### Cell culture

Bone marrow was isolated from the tibias of donor mouse strains C57BL/6, *MyD88*^*-/-*^, *RIPK3*^*-/-*^ and *RIPK3*^*-/-*^*Caspase8*^*-/-*^. A 10 ml syringe loaded with a 27 Ga needle was used to flush the bone marrow out of the tibias into a 50 ml conical tube. The media containing the bone marrow was subsequently passed through an 18 Ga needle. The bone marrow suspension was plated into five petri dishes, and cells were allowed to differentiate into bone marrow derived macrophages (BMDM) in DMEM (VWR) supplemented with 10% BGS (HyClone), nonessential amino acids (ThermoFisher Scientific), 100 U/mL penicillin (ThermoFisher Scientific), 0.1 mg/mL streptomycin (ThermoFisher Scientific) and L929 culture supernatant as previously described [42]. BMDM were harvested after five days of differentiation for use in experiments.

Peritoneal exudate cells (PEC) were collected by filling the peritoneal cavity with sterile PBS then using a 21 Ga needle to collect the PBS containing PEC. The cells were re-suspended in cDMEM consisting of DMEM (VWR) supplemented with 10% bovine growth serum (HyClone), 1% sodium pyruvate (ThermoFisher Scientific), 1% Non-Essential Amino Acids (ThermoFisher Scientific), 3% HEPES (ThermoFisher Scientific), 0.0001% 2-mercaptoethanol (Sigma, St. Louis, MO), 100 U/ml penicillin (ThermoFisher Scientific) and 0.1 mg/ml streptomycin (ThermoFisher Scientific). Splenic single cell suspensions were prepared by homogenizing spleens, then lysing red blood cells with RBC ACK lysis buffer (ThermoFisher Scientific) as previously described [33]. Removal of cell debris was accomplished by filtration through a 40 μm cell strainer. Cells were washed in PBS and re-suspended cDMEM. Mesenteric lymph node (MLN) single cell suspensions were prepared by crushing the tissue through a 40 μm cell strainer. The cells were re-suspended in cDMEM. Cells were stimulated with soluble tachyzoite lysate antigen (STAg) and supernatants were collected for cytokine ELISA 72 hr later. STAg was prepared from RH strain tachyzoites by sonication of parasites at 0°C in the presence of protease inhibitors, followed by centrifugation at 10,000 x g. Supernatant was collected and dialyzed into PBS and stored in aliquots at -80°C.

### *In Vivo* IL-12 depletion

Mice were i. p. injected with 0.5 mg anti-mouse IL-12p40 mAb (BioXCell, New Hampshire, Catalog #BE0051) or normal rat gamma globulin (Jackson Immunoresearch, West Grove PA, Catalog #012000002) at Day 0. On Day 1, mice were i. p. inoculated with 5 x 10^6^ *cps1-1* or *cps1-1*:*Δgra24* tachyzoites. On Days 3 and 6, mice received further injections with anti-IL-12 mAb or control Ab (0.5 mg per mouse). On Day 11 splenocytes were isolated for *in vitro* cytokine assay.

### Flow cytometry

Single cell suspensions of peritoneal exudate cells were stained with primary antibodies including anti-CD11c eFluor610 (Catalog # 61011482, eBioscience, San Diego, CA), anti-MHCII AlexaFluor 647 (Catalog # 107618, BioLegend, San Diego, CA), anti-F4/80 Brilliant Violet 711 (Catalog # 123147, BioLegend), and anti-Ly-6G PE/Cy7 (Catalog #127618, BioLegend) for 20 min at 4°C in FACS buffer (1% BGS/ 0.01% NaN_3_ in PBS). Cells were then fixed in 3.7% formaldehyde (MilliporeSigma, Burlington, MA) for 15 min and then permeabilized in permeabilization buffer (0.1% saponin in PBS). Intracellular *T*. *gondii* tachyzoites were detected by staining with monoclonal anti-*T*. *gondii* (TP3) AlexaFluor 700 (Catalog # NB1102570, Novus Biologicals, Centennial, CO) for 45 min at 4°C in permeabilization buffer. All samples were run on a four laser (violet, blue, yellow, red) Attune NxT flow cytometer (ThermoFisher Scientific) and the data were analyzed using FlowJo v.10 software (FlowJo, Ashland, OR).

### Cytokine ELISA

The production of IL-12p40 and IFN-γ were measured using a murine IL-12/IL-23p40 ELISA Kit (Invitrogen, Carlsbad, CA) and a IFN-γ ELISA Kit (Invitrogen) according to the manufacturer’s instruction. Briefly, 96 well ELISA plates (Corning Costar, Corning, NY) were coated with anti-cytokine capture antibody in coating buffer (Invitrogen) and incubated overnight at 4°C. The plates were washed 3 times in PBS containing 0.05% Tween-20 (PBST) then blocked with diluent (Invitrogen) for 1 hr at room temperature. After washing the plates once in PBST, sample supernatants as well as recombinant standard was added and incubated overnight at 4°C. The plates were washed 5 times in PBST then anti-cytokine biotin detection antibody was added and plates were incubated at room temperature for 1 hr. The plates were washed 5 times in PBST, avidin-horseradish peroxidase was added and plates were incubated for 30 min at room temperature. The plates were washed 5 times in PBST and 3,3’,5,5’-Tetramethylbenzidine (TMB) was added and plates incubated for 20 min. The reaction was quenched with 2 M H_2_SO_4_ and plates were read at 450 nm on an iMark ELISA reader (Bio-Rad, Hercules, CA).

### Western blot analysis

BMDM were plated overnight in a 24-well tissue culture plate, then parasites were added at a 3: 1 ratio of tachyzoites to cells. The plates were centrifuged at 63 x g for 3 min to initiate contact between parasites and cells. Lysates were collected using 200 μl SDS lysis buffer and passaged 3 times through a 27 Ga needle to shear DNA. The samples were boiled for 5 min and subjected to Western blot analysis using primary anti- phospho-p38 antibody (Catalog #4511, Cell Signaling, Danvers, Massachusetts), anti-phosphoERK1/2 (Catalog #4370, Cell Signaling), and anti-phosphoSTAT3 (Catalog #4074, Cell Signaling). Samples were also probed for total p38 (Catalog #9218, Cell Signaling), total ERK1/2 (Catalog #4695, Cell Signaling) and total STAT3 (Catalog #4904, Cell Signaling). Lysates were loaded into a 10% acrylamide protein gel (Bio-Rad). Proteins were transferred onto a nitrocellulose membrane then blocked with Tris-buffered PBS containing 5% nonfat dry milk and 0.05% Tween 20 for 2 hr. The nitrocellulose membranes were washed 3 times before the primary antibody was added in Tris-buffered PBS containing 5% BSA and 0.05% Tween 20 for overnight incubation on a rocking platform at 4°C. The nitrocellulose membranes were washed 6 times, then horseradish peroxidase-linked anti-rabbit antibody was added and membranes were incubated for 2 hr at room temperature on a rocking platform. A chemiluminescent substrate system (ThermoFisher Scientific) was used to detect the bound antibodies.

### Cytokine proteome array

Cytokine and chemokine expression was measured using the Murine Proteome Profiler Mouse XL Cytokine Array (R&D Systems, Minneapolis, MN). To obtain samples, C57BL/6 mice were i. p. inoculated with either *cps1-1* or *cps1-1*:*Δgra24* (10^**6**^ tachyzoites per mouse). Four days post infection the peritoneal exudate cells were collected by filling the peritoneal cavity with sterile PBS then using a 21 Ga needle to collect the cell suspension. The PECs were plated in cDMEM at 2 x 10^**6**^ cells per well and were incubated for 72 hr. Following incubation, the supernatants were collected and the Murine Proteome Profiler Mouse XL Cytokine Array was used according to the manufacturer’s directions. In brief, nitrocellulose membranes were blocked for 1 hr then sample supernatants were added to the membranes. After overnight incubation, membranes were washed and a detection antibody cocktail (R&D Systems) was added. The membranes were incubated with the detection antibody for 1 hr, washed, and streptavidin-horseradish peroxidase was added. After 30 min incubation, membranes were washed three times (10 min per wash) and spots visualized by addition of enhanced chemiluminescence reagent. The membranes were imaged on a Chemi Touch Imaging System (Bio-Rad), and semi-quantitative analysis was accomplished using ImageJ software.

### qPCR for parasite load

DNA was extracted from tissues using the DNeasy Blood and Tissue Kit (Qiagen Inc. Valencia, CA) following the manufacturer’s instruction. Quantitative PCR was performed on the indicated tissue targeting the conserved *Toxoplasma* B1 gene and the murine argininosuccinate lyase (ASL) gene [21]. The extracted DNA from each tissue was normalized to 125 ng of DNA for each 20 μl reaction. A 5 μM primer working solution containing the forward and reverse primers for B1 (forward 5’-GGA-GGA-CTG-GCA-ACC-TGG-TGT-CG-3’, reverse 5’-TTG-TTT-CAC-CCG-GAC-CGT-TTA-GCA-3’) and a 5 μM working solution containing the forward and reverse primers for ASL (forward 5’-TCT-TCG-TTA-GCT-GGC-AAC-TCA-CCT-3’, reverse 5’-ATG-ACC-CAG-CAG-CTA-AGC-AGA-TCA-3’) was made. 2.5 μl of primer solution of B1 or ASL was added to 10 μl SYBR green (Bio-Rad), 2.5 μl molecular biology grade water and 4 μl of template for a total volume of 20 μl. The reactions were carried out using a Bio-Rad CFX96 Real Time System C1000 Touch thermal cycler with the following thermal cycling conditions: 98°C for 5 min, 95°C for 5 sec, 60°C for 30 sec followed by 40 cycles of 95°C at 5 sec and 60°C for 30 sec. A melting curve analysis was performed to ensure specificity of amplification. Quantification of the number of B1 and ASL gene copy numbers was accomplished by using a quantitative standard curve. Bio-Rad CFX manager version 3 software was used to quantify B1 and ASL gene copy number. Non-infected tissues were used as negative controls and molecular grade water was used as a negative template control.

### Immunofluorescence

HFF were grown to confluency on glass coverslips for five days. The confluent HFFs were infected with tachyzoites in 1% HFM in the presence or absence of uracil (0.3 mM). After 48 hrs, coverslips were washed in PBS, then fixed with 3.7% PFA for 20 min. The cells were washed in permeabilization buffer (PB; 0.1% saponin in PBS) then blocked in PB containing 5% normal mouse serum (Invitrogen) for 1 hour. After blocking, the cells were washed with PBS and stained by the addition of goat anti-*Toxoplasma* FITC-conjugated antibody (Catalog #PA17253, Invitrogen) and Texas Red-X phalloidin (Catalog #T7471, Invitrogen) diluted in PB containing 5% bovine serum albumin (VWR) for 1 hr. The cells were washed 3 times in PB and 4 times in PBS. Finally, the coverslips were dried then mounted onto slides using DAPI containing mounting media (Catalog #P36962, Invitrogen). Imaging was accomplished with a BX53 fluorescence microscope (Olympus America, Inc, Waltham, MA) and DP manager software (Olympus).

### Quantification of Immunofluorescence staining

For the quantification of percent infection, 100–150 cells were counted per field of view (n = 10 fields per treatment). Infected cells were counted and percent infection was calculated. To quantify average number of tachyzoites per vacuole, number of parasites in each parasitophorous vacuole (20–30 per field of view) was counted and average number of tachyzoites per vacuole was calculated.

### Statistical analyses

Student t-test was used to assess the significant difference between groups. A p value <0.05 was considered significant. A two way ANOVA with a Tukey’s multiple comparisons test was used to compare three groups across multiple time points. A Mann-Whitney test was used to assess statistical significance in the *in vivo* IL-12 depletion experiment. Survival after challenge with a virulent RH strain was assessed using a Kaplan-Meier curve and the statistical significant difference between groups was calculated using the Log-Rank test with the use of GraphPad Prism Software. All experiments were repeated 2–3 times.

## Supporting information

S1 Fig IL-12 responses to *Toxoplasma* strains PTG and RH in WT and MyD88 KO BMDM. BMDM were generated from C57BL/6 (**A**) and *MyD88*^*-/-*^ (**B**) mice and infected with either Type I RH or Type II PTG tachyzoites at a 1:1 ratio of parasites to cells. Supernatants were collected for cytokine ELISA at the indicated time points. NI, non-infected BMDM, supernatants were collected at 24 hr. Data shown are the means ± SD of cells cultured in triplicate. Statistical significance was analyzed using two way ANOVA with Tukey's multiple comparisons test (*** p > 0.001). This experiment was repeated two times and yielded similar results.
(TIF)

S2 Fig Caspase 8 is not involved in *Toxoplasma*-induced IL-12 production in BMDM. BMDM were generated from C57BL/6 (WT), *RIPK3^-/-^, RIPK^-/-^caspase8^-/-^* mice and infected with either Type I RH (A) or Type II PTG (B) tachyzoites at a 1:1 ratio of parasites to cells. Supernatants were collected for cytokine ELISA at the indicated time points. NI, non-infected BMDM, supernatants were collected at 24 hr. Data shown are the means ± SD of cells cultured in triplicate, n = 2 mice per group. Statistical significance was assessed using two way ANOVA with Tukey's multiple comparisons test (** p > 0.01).
(TIF)

S3 Fig GRA24 does not control phosphorylation of ERK1/2 or phosphorylation of STAT3 triggered by *Toxoplasma*. BMDM from *MyD88*^*+/+*^ and *MyD88*^*-/-*^ mice were infected with *cps1-1* or *cps1-1*:*Δgra24* tachyzoites at a 1:1 ratio of parasites to cells. Cell lysates were prepared for Western blot analysis at the indicated time points.
(TIF)

S4 Fig Cytokine proteome array coordinates. Diagram indicating the coordinates of each cytokine and chemokine included on the cytokine proteome array.
(TIF)
