# Supplementary figures and images for: Toxoplasma gondii dense granule protein GRA24 drives MyD88-independent p38 MAPK activation, IL-12 production and induction of protective immunity
Source: PLoS Pathog. 2020 May 15;16(5):e1008572. doi: 10.1371/journal.ppat.1008572 (PMC7255617; doi:10.1371/journal.ppat.1008572)

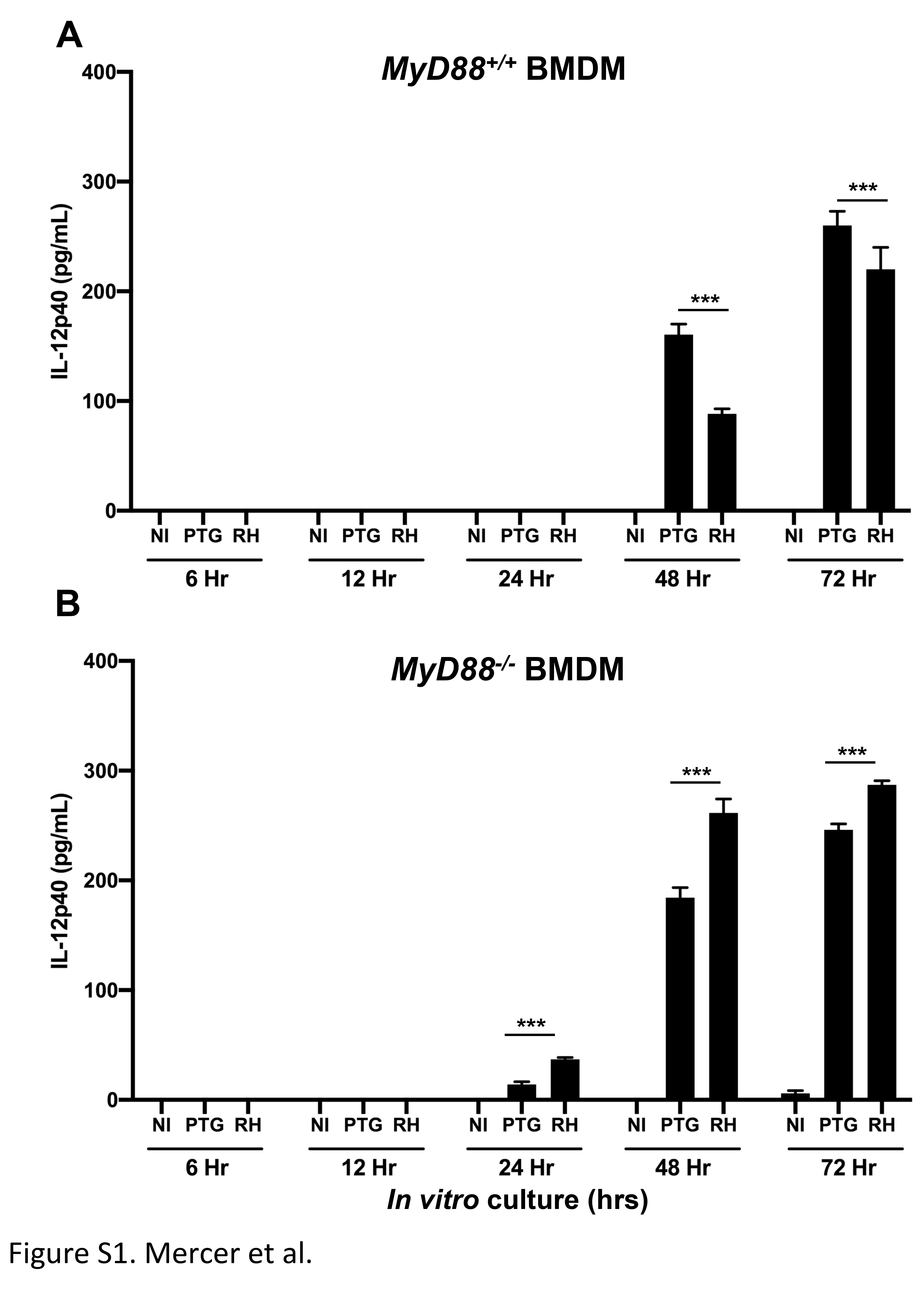

Supplement: S1 Fig — BMDM were generated from C57BL/6 (A) and MyD88-/- (B) mice and infected with either Type I RH or Type II PTG tachyzoites at a 1:1 ratio of parasites to cells. Supernatants were collected for cytokine ELISA at the indicated time points. NI, non-infected BMDM, supernatants were collected at 24 hr. Data shown are the means ± SD of cells cultured in triplicate. Statistical significance was analyzed using two way ANOVA with Tukey's multiple comparisons test (*** p > 0.001). This experiment was repeated two times and yielded similar results. (TIF) [file ppat.1008572.s001.tif]

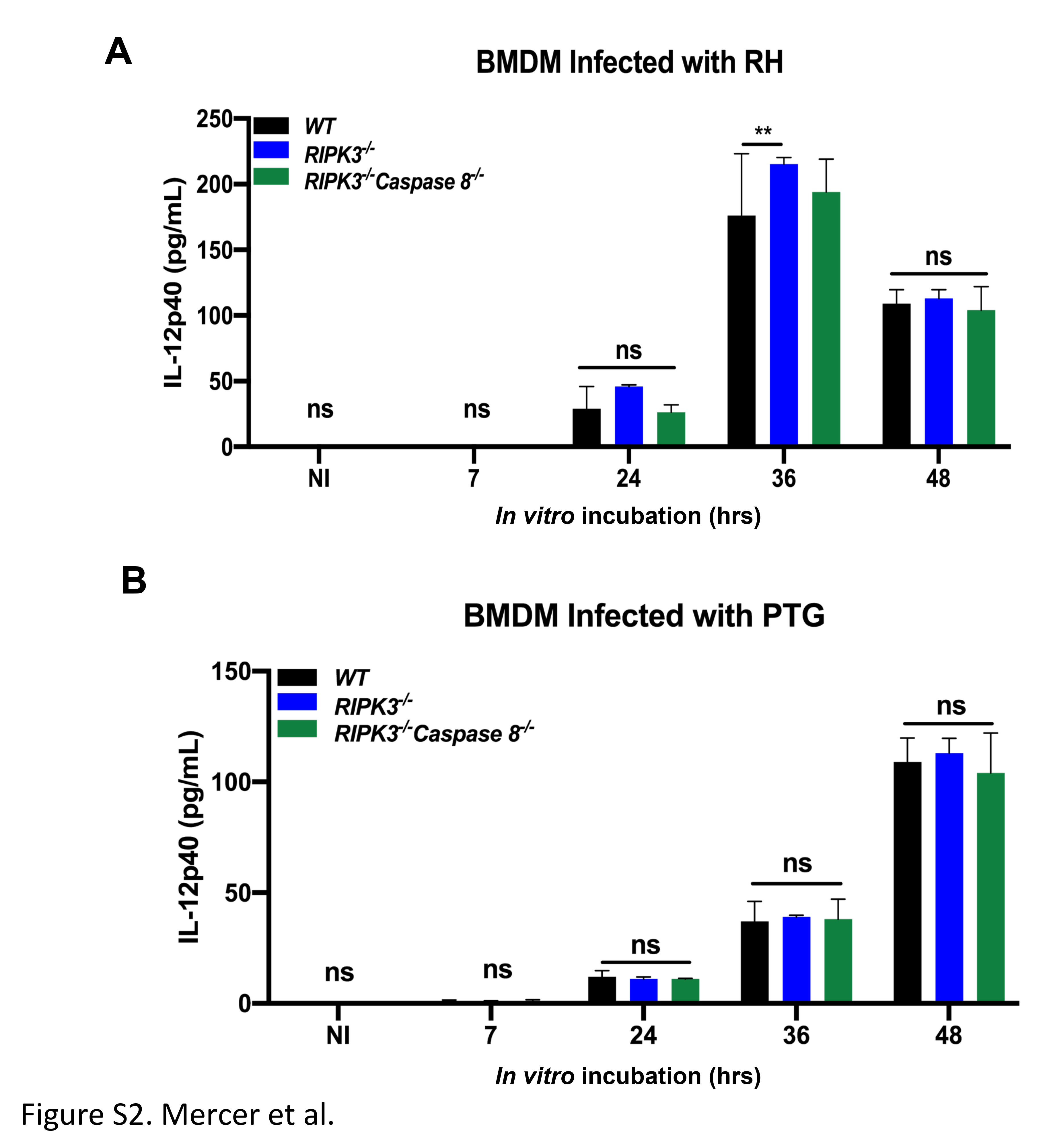

Supplement: S2 Fig — BMDM were generated from C57BL/6 (WT), RIPK3-/-, RIPK-/-caspase8-/- mice and infected with either Type I RH (A) or Type II PTG (B) tachyzoites at a 1:1 ratio of parasites to cells. Supernatants were collected for cytokine ELISA at the indicated time points. NI, non-infected BMDM, supernatants were collected at 24 hr. Data shown are the means ± SD of cells cultured in triplicate, n = 2 mice per group. Statistical significance was assessed using two way ANOVA with Tukey's multiple comparisons test (** p > 0.01). (TIF) [file ppat.1008572.s002.tif]

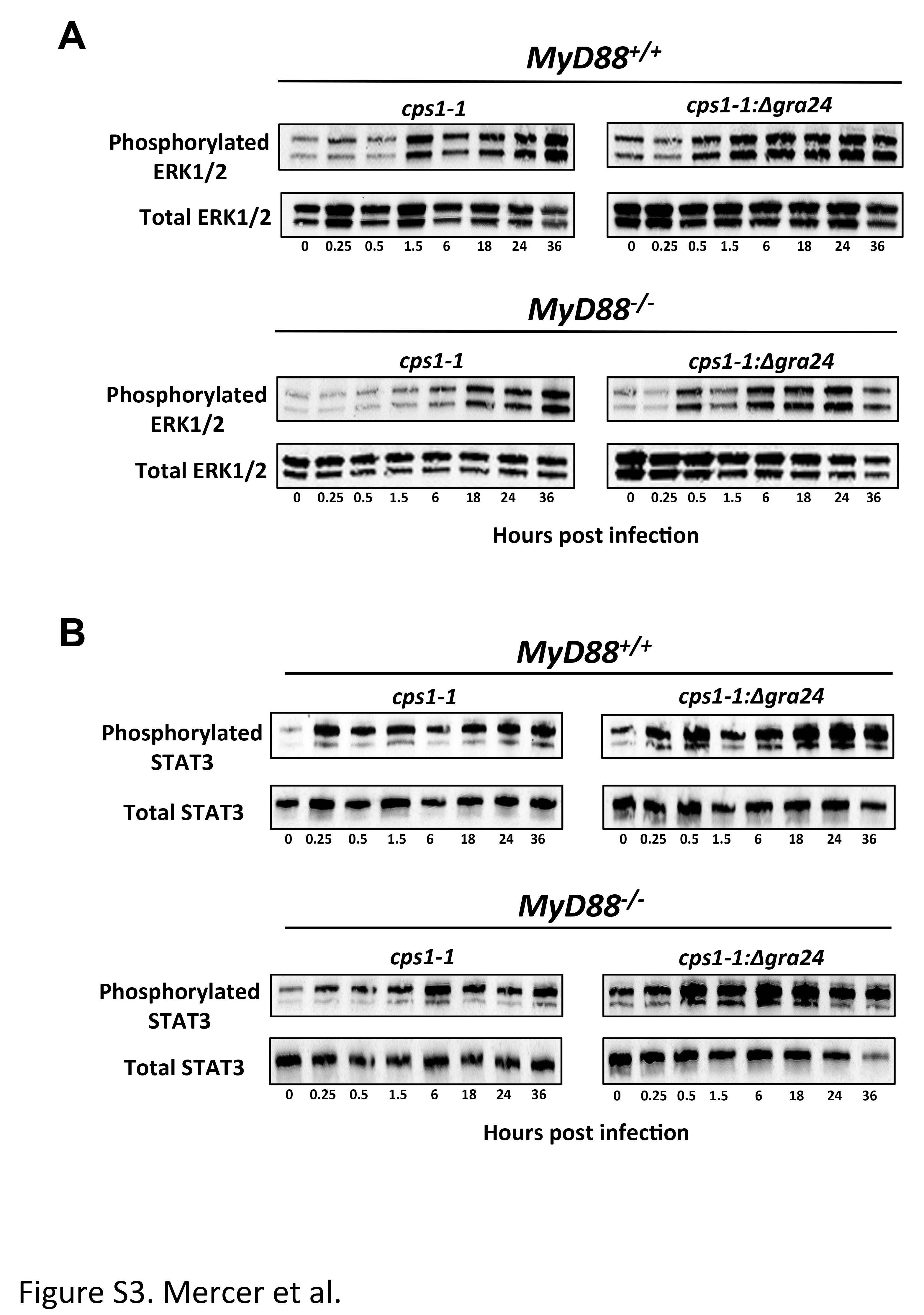

Supplement: S3 Fig — BMDM from MyD88+/+ and MyD88-/- mice were infected with cps1-1 or cps1-1:Δgra24 tachyzoites at a 1:1 ratio of parasites to cells. Cell lysates were prepared for Western blot analysis at the indicated time points. (TIF) [file ppat.1008572.s003.tif]

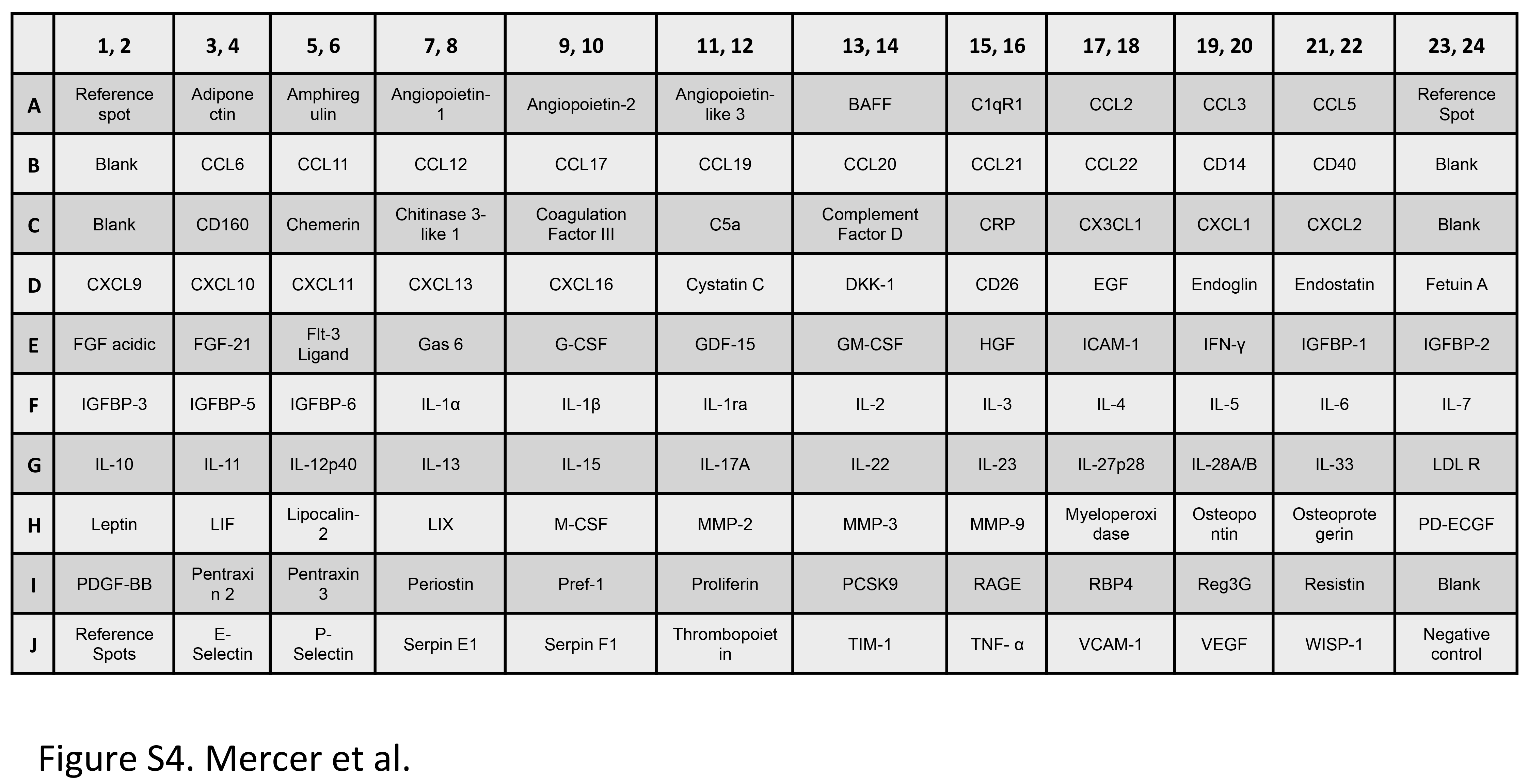

Supplement: S4 Fig — Diagram indicating the coordinates of each cytokine and chemokine included on the cytokine proteome array. (TIF) [file ppat.1008572.s004.tif]
